# Increased Mitochondrial Fragmentation Mediated by Dynamin-Related Protein 1 Contributes to Hexavalent Chromium-Induced Mitochondrial Respiratory Chain Complex I-Dependent Cytotoxicity

**DOI:** 10.3390/toxics8030050

**Published:** 2020-07-29

**Authors:** Yu Ma, Yujing Zhang, Yuanyuan Xiao, Fang Xiao

**Affiliations:** Department of Health Toxicology, Xiangya School of Public Health, Central South University, Changsha 410078, China; 196901002@csu.edu.cn (Y.M.); zhangyujing@hunnu.edu.cn (Y.Z.); xiaoyuanyuan@csu.edu.cn (Y.X.)

**Keywords:** hexavalent chromium [Cr(VI)], mitochondrial fragmentation, dynamin-related protein 1 (Drp1), mitochondrial respiratory chain complex I (MRCC I), reactive oxygen species (ROS)

## Abstract

Hexavalent chromium (Cr(VI)) pollution is a severe public health problem in the world. Although it is believed that mitochondrial fragmentation is a common phenomenon in apoptosis, whether excessive fission is crucial for apoptosis remains controversial. We previously confirmed that Cr(VI) mainly targeted mitochondrial respiratory chain complex I (MRCC I) to induce reactive oxygen species (ROS)-mediated apoptosis, but the related mechanism was unclear. In this study, we found Cr(VI) targeted MRCC I to induce ROS accumulation and triggered mitochondria-related cytotoxicity. Cr(VI)-induced cytotoxicity was alleviated by pretreatment of Glutamate/malate (Glu/Mal; MRCC I substrates), and was aggravated by cotreatment of rotenone (ROT; MRCC I inhibitor). Cr(VI) induced excessive mitochondrial fragmentation and mitochondrial dynamin-related protein 1 (Drp1) translocation, the application of Drp1-siRNA alleviated Cr(VI)-induced apoptosis. The cytotoxicity in the Drp1-si plus Cr(VI) treatment group was alleviated by the application of Glu/Mal, and was aggravated by the application of ROT. Drp1 siRNA promoted the inhibition of Glu/Mal on Cr(VI)-induced cytotoxicity, and alleviated the aggravation of ROT on Cr(VI)-induced cytotoxicity. Taken together, Cr(VI)-induced Drp1 modulation was dependent on MRCC I inhibition-mediated ROS production, and Drp1-mediated mitochondrial fragmentation contributed to Cr(VI)-induced MRCC I-dependent cytotoxicity, which provided the experimental basis for further elucidating Cr(VI)-induced cytotoxicity.

## 1. Introduction

Chromium (Cr) widely exists in the ecological environment and can be found in pigments, chrome-plated metals, cement, detergents, and industrial Cr waste dumps [1]. Cr has a variety of oxidation states (−2 to +6), but only trivalent chromium (Cr(III)) and hexavalent chromium (Cr(VI)) are stable. The increase in industrial use, coupled with improper disposal of Cr(VI)-related waste, has led to the serious increase of Cr(VI) levels in air, water, and soil, resulting in the pollution of the environment, even the food chain [2].

Mitochondria constantly undergo a dynamic fusion/fission process, which is mainly controlled by regulatory proteins such as mitofusins (Mfns) and dynamin-related protein 1 (Drp1) [3]. This kind of dynamic balance is essential to maintain constant changes in mitochondrial shape, size, and network. Mitochondrial fission process is mainly mediated by Drp1, which exists in the cytosol and translocates to the outer membrane of mitochondrial during fission [4]. Increasing evidence suggested that the dynamic morphology of the mitochondrial network is very important. In the physiological state, the long, continuous tracks of fused mitochondria and branching networks are dominant and mainly regulated by Mitofusins (Mfns). However, upon exposure to various stresses, mitochondria undergo fission, networks become unraveled, and the fragmented morphology is more prominent (regulated Drp1). Preliminary studies have shown that Drp1 ablation declined Cyt c release and inhibited apoptosis [5]. In contrast, other studies revealed that blockage of Drp1 partially decreased [6] or had little effect [7] on Cyt c release, without affecting apoptosis.

The electron transport chain (ETC), which exists in the folded inner membranes of mitochondria, is mainly composed of four mitochondrial respiratory chain complexes (MRCC; I–IV) and two free-moving electron transfer carriers cytochrome c (cyt c) and ubiquinone. The four complexes are assembled into a specifically configured super-complex, which together with MRCC V (F1F0ATP synthase), becomes the basis of ATP generation during oxidative phosphorylation [8]. As the largest multi-subunit enzyme complex located in ETC, MRCC I is also called NADH-ubiquinone oxidoreductase, and its key role is to transfer electrons from NADH to ubiquinone [9]. The ETC is the key component of mitochondria and also known as the most important source of intracellular reactive oxygen species (ROS). ROS include oxygen-free radicals such as hydroxyl radical (OH) and superoxide anion radical (O_2_^−^), and non-radical oxidants such as hydrogen peroxide (H_2_O_2_). Due to the existence of electron leakage, not all the electrons could be successfully transferred to the final electron acceptor, O_2_. Under normal conditions, 0.2–2% of the electrons do not follow the transmission order but directly leak out from ETC, and then interact with oxygen to produce ROS [10]. As a double-edged sword, ROS plays an important role in intracellular signaling pathways, but ROS accumulation can lead to cytotoxicity and even cell death.

Although it is believed that mitochondrial fragmentation is a common phenomenon in apoptosis, whether excessive fission is crucial for apoptosis progression remains controversial. Our previous studies [11,12] have demonstrated that Cr(VI) mainly targeted MRCC I and increased ROS generation to induce cytotoxicity. In addition, Cr(VI) could also cause both mitochondrial damage and apoptotic cell death during ROS-triggered cytotoxicity, but the related mechanism involved in MRCC I-dependent cytotoxicity was unclear. The present study will demonstrate the role of increased mitochondrial fragmentation mediated by Drp1 in Cr(VI)-induced MRCC I-dependent cytotoxicity, which will provide experimental evidence for further elucidating the cytotoxicity of Cr(VI).

## 2. Materials and Methods

### 2.1. Cell Culture and Cell Counts

Human L02 hepatocytes, obtained from Experimental Central of Xiangya Hospital of Central South University, Changsha, China, were cultured in 25 cm^2^ culture flasks in the standard humidified incubator with the set of 5% CO_2_ and 37 °C. Roswell Park Memorial Institute (RPMI) 1640 medium supplemented with 10% fetal bovine serum (FBS) (Gibco, Carlsbad, CA, USA) and 1% penicillin-streptomycin (P/S) solution was used.

Under the optimal conditions, the doubling time of L02 hepatocytes was 18–24 h, and the cells were subcultured every 2.5–3 days (d). Cell numbers were measured and recorded everyday using a hemocytometer by trypan blue exclusion method.

### 2.2. Drp1 SiRNA

The siRNA sequences were designed and synthesized by RibobioCo. Ltd. (Guangzhou, China). The hepatocytes were transfected with siRNA targeting Drp1 (siB121119100350-1-5) and its negative control (siB06525141910-1-5) using lipofectamine 3000 (Invitrogen, Carlsbad, CA, USA). After 4 h of transfection, the hepatocytes were changed with complete medium.

### 2.3. ROS Level

The intracellular ROS level was determined using 2′, 7′-dichlorofluorescein diacetate (DCFH-DA; Beyotime Institute of Biotechnology, Shanghai, China) by flow cytometry. L02 hepatocytes were treated with indicated chemicals and washed twice by cold PBS and loaded with 10 µM DCFH-DA at 37 °C for 40 min. After the incubation, the hepatocytes were washed again and analyzed by flow cytometry.

### 2.4. Alanine Aminotransferase (ALT) and Aspartate Aminotransferase (AST) Levels

L-02 hepatocytes were treated with different chemicals, and the supernatants were collected. ALT and AST levels were determined using the commercial kits (Jiancheng, Nanjing, China) according to the instructions. The optical density at 510 nm was measured using the multifunctional microplate reader.

### 2.5. Caspase-3 Activity

Caspase-3 activity was determined using the commercial colorimetric assay kit. The hepatocytes were treated with indicated chemicals and washed twice with PBS, followed by lysis with the Caspase-3 Assay kit (Beyotime Institute of Biotechnology, China). The centrifugation was performed at 16,000× *g* at 4 °C for 10 min. The samples were then incubated with Ac-DEVD-pNA (substrate) at 37 °C for 2 h. The absorbance was measured and recorded by a microplate reader at 405 nm.

### 2.6. Apoptosis

The cell apoptosis was determined using a commercial Annexin V-FITC apoptosis detection kit (Invitrogen, Carlsbad, CA, USA). After the treatment of different chemicals, the hepatocytes were incubated with 100 μL 1× binding buffer containing 5 μL Annexin-V-FITC and 1 μL propidium iodide (PI) for 30 min at room temperature. After the incubation, 400 μL binding buffer was added to the culture to stop the staining. The flow cytometric analysis was then performed. Data was analyzed using Flowjo 7.6 software.

### 2.7. Mitochondrial Permeability Transition Pore (mPTP) Opening

The mPTP opening was examined using the commercial kit by monitoring the release of calcein from mitochondrial. Briefly, the hepatocytes were treated with 2 µM calcein-AM and 1 mM CoCl2 for 30 min at room temperature, and washed with PBS. The culture was then incubated with 1 mM CoCl2 for an additional 20 min at 37 °C in order to specifically quench the fluorescence of free calcein in the cytosol. The fluorescence intensity of mitochondrial calcein in L02 hepatocytes was determined using a fluorescence microplate reader at 490 nm/515 nm for excitation/emission. The loss of calcein fluorescence suggested the opening of mPTP.

### 2.8. Mitochondrial Membrane Potential (MMP, Δψm)

The MMP of L02 hepatocytes was detected using JC-1 (Sigma, St. Louis, MO, USA). After the chemicals treatment, the cells were washed with PBS and then incubated with JC-1 for 20 min at 37 °C. The fluorescence intensity was read at the excitation/emission wavelength of 488/530 nm. The MMP is presented as % of control for the fluorescence intensity.

### 2.9. qRT-PCR

Total RNA was isolated with the TRIzol reagent (Invitrogen, Carlsbad, CA, USA) following the manufacturer’s instruction. The RNA quality was verified by spectrophotometry. Later on, the cDNAs were synthesized using the ReverTra Ace qPCR RT kit (Toyobo, Tokyo, Japan). For mitochondrial DNA analyses, total DNA was extracted by a DNA extraction kit (NEP002-1, Dingguo, China) according to the manufacturer’s instructions. qRT-PCR was performed using the Light Cycler^®^Nano SYBR Green I Master on a Light Cycler^®^ Nano System. The PCR conditions were as follows: 10 min at 95 °C, followed by 40 cycles of 95 °C for 30 s, 56 °C for 30 s and 72 °C for 30 s. The mRNA levels were calculated using the 2−ΔΔCT method normalized to ACTB mRNA. 

The Primers used in this study: Drp1, 5′-TAGTGGGCAGGGACCTTCTT-3′ (F) and 5′-TGCTTCAACTCCATTTTCTTCTCC-3′ (R); ACTB, 5′-CACCAGGGCGTGATGGT-3′ (F) and 5′-CTCAAACATGATCTGG GTCAT-3′ (R); NADH dehydrogenase subunit I (ND1), 5′-TACGCAAAGGTTCCCAACG-3′ (F) and 5′-GGTGATGGTGGATGTGGC-3′ (R); cytochrome C Oxidase Subunit IV Isoform 1 (COX4I1), 5-TAGAAACCGTCTGAACTATCC-3′ (F) and 5′- ATGATTATGAGGGCGTGA-3′ (R); β-globin, 5′-GTTACTGCCTG TGGGGCAA-3′ (F) and 5′-CAAAGGTGCCCTTGAGGTT-3′ (R). The qRT-PCR assay was designed and performed in accordance with the Minimum Information for Publication of Quantitative Real-Time PCR Experiments (MIQE) guidelines.

### 2.10. Mitochondria Mass

Briefly, after the treatment of different chemicals, the hepatocytes were exposed to Mito-Tracker Green (10 μM, 30 min) at 37 °C in the dark. Then the cells were thoroughly washed with pre-warmed PBS, and analyzed with flow cytometer at 490/516 nm for excitation/emission wavelengths.

### 2.11. ATP Level

The ATP level was examined using a luciferase-based luminescence enhanced ATP assay kit (Beyotime Institute of Biotechnology, China) following the manufacturer’s instruction. The hepatocytes were washed with ice-cold PBS, lysed with 200 μL lysis buffer, and then centrifuged at 12,000× *g* for 5 min at 4 °C. ATP content in cell lysates was then determined using a luminescence plate reader.

### 2.12. Western Blotting

Mitochondrial fraction was isolated using the Mitochondria Isolation Kit for Cultured Cells according to the manufacturer’s instructions (Beyotime Institute of Biotechnology, Nanjing, China). For total protein extraction, cells were washed twice with PBS and lysed with RIPA buffer (Beyotime Institute of Biotechnology, Nanjing, China). The homogenates were centrifuged at 12,000× *g* for 15 min at 4 °C, the supernatant was then collected. Protein concentrations were evaluated using the BCA method. Then the samples were denatured by boiling with sample buffer for 10 min, loaded to SDS-PAGE gel for separation, and then transferred onto the PVDF membrane. The membranes were blocked with 5% skim milk for 1 h at room temperature and incubated with different primary antibodies overnight at 4 °C. The membranes were then washed and incubated with secondary antibodies for 1 h at room temperature. The protein bands were visualized using the enhanced chemiluminescence (ECL) kit (Thermo, Waltham, MA, USA) and quantitated using Image J software (National Institutes of Health, USA). The band density of different proteins was normalized to the control.

ND1 (DF4214) antibody was obtained from Affinity Biosciences (Cincinnati, OH, USA). VDAC1 (55259-1-AP) antibody was purchased from Proteintech Group Inc. (Wuhan, China). Drp1 (A2586), AIF (A19536), cyt c (A4912), and caspase-3 (A11021) antibodies were purchased from ABclonal Technology (Wuhan, China). Antibody against β-actin (70-ab008-040) was obtained from MultiSciences Biotech Co. (Hangzhou, China).

### 2.13. MRCCs Activity

After the treatment, L02 hepatocytes were collected and suspended in 0.1 M phosphate buffer (pH 7.2). To ensure cellular disruption and after three cycles of freeze/thawing, the activities of MRCC I–V were determined spectrophotometrically as previously described [11].

### 2.14. Confocal Microscope

For mitochondrial morphology examination, the hepatocytes were incubated with 10 nM Mitotracker Red (Invitrogen Life Technologies, Carlsbad, CA, USA) for 45 min at 37 °C. After the indicated chemicals treatment, the hepatocytes were incubated with DAPI for another 45 min at 37 °C in the dark. The fluorescence images of each group were captured using Leica TCS SP5 II confocal spectral microscope. More than 20 clearly identifiable mitochondria were randomly selected from each treatment group. The length and density of the mitochondria were analyzed using Image J software.

To analyze Drp1 mitochondrial translocation, Cr(VI)-exposed hepatocytes were fixed with 4% paraformaldehyde for 15 min at room temperature following incubation with MitoTracker Red (10 nM, 45 min, 37 °C). The hepatocytes were then permeabilized with 0.5% Triton X-100, blocked with 5% bovine serum albumin (BSA), and incubated with primary Drp1 antibody at a 1:100 dilution at 4 °C overnight, followed by treatment with the secondary antibody. Nuclei were stained with DAPI prior to mounting. Confocal fluorescence images of each treatment group were captured with the Leica TCS SP5 II confocal spectral microscope.

### 2.15. Statistical Analysis

All experiments were repeated at least three times. Representative experiments or mean ± SD are shown in the figures. Statistical analyses were performed using one-way of variance (ANOVA), Student’s *t*-test, using SPSS 17.0 software. A significant difference was taken as *p* < 0.05.

## 3. Results

### 3.1. MRCC I Inhibition Led to Cytotoxicity in L02 Hepatocytes

**Figure 1:**
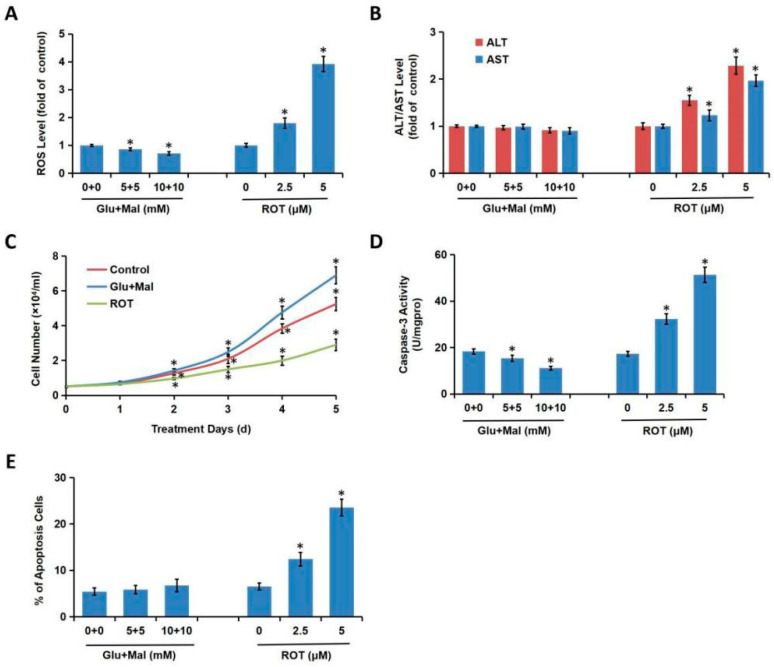
MRCC I inhibition led to cytotoxicity in L02 hepatocytes. (**A**) The L02 hepatocytes were either exposed to different concentrations of MRCC I substrates Glu/Mal (0, 5/5, 10/10 mM) for 1 h and then cultured for 23 h, or exposed to different concentrations of ROT (0, 2.5, 5 μM) for 24 h. ROS level was determined using the fluorescent probe DCFH-DA. (**B**) AST/ALT level was determined using the related kits. (**C**) The L02 hepatocytes were cultured for consecutive 5 days and treated with various concentrations of Glu/Mal (0, 5/5, 10/10 mM; 1 h) and ROT (0, 2.5, 5 μM; 24 h) in day 0 and day 3. The cell number, which indicated growth and proliferation of the hepatocytes, was recorded using a hemocytometer by trypan blue exclusion method. (**D**) Caspase-3 activity was examined using the commercial colorimetric assay kit. (**E**) The cell apoptosis was determined using commercial Annexin V-FITC Apoptosis Detection Kit. * *p* < 0.05, compared with the control group.

Glutamate/malate (Glu/Mal), known as the substrates of MRCC I, can directly initiate the mitochondrial main respiratory chain (NADH respiratory chain) by activating MRCC I. The inhibitor of MRCC I is rotenone (ROT). We first evaluated whether changes in MRCC I activity would affect cytotoxicity. The L02 cells were treated with different concentrations of Glu/Mal (0, 5/5, 10/10 mM; 1 h) and ROT (0, 2.5, 5 μM; 24 h). As shown in Figure 1A, the application of substrates Glu/Mal slightly inhibited ROS generation, while the application of the inhibitor ROT significantly enhanced ROS generation in a concentration-dependent manner. In most cases, the degree of ALT and AST increase was consistent with the degree of hepatocyte damage, which is the most commonly used indicator of hepatocytes/liver function. The distribution of these two enzymes in hepatocytes is different. ALT is mainly distributed in the cytoplasm, and the increase of ALT leakage indicates the membrane damage. AST is mainly distributed in both cytoplasm and the mitochondria, and the increase of ALT indicates that hepatocytes are damaged to organelle level. Compared with the control group, the treatment of Glu/Mal did not alter the leakage of ALT/AST to the culture medium, while the application of ROT increased the leakage of these two enzymes (especially ALT) in a concentration-dependent manner (Figure 1B). We further examined whether the alteration of MRCC I activity would affect cell growth and proliferation. As shown in Figure 1C, from day 2 and compared with control, Glu/Mal increased while ROT significantly decreased cell number, indicating that Glu/Mal stimulated while ROT suppressed cell growth and proliferation. Caspase-3 activity was inhibited by Glu/Mal and increased by ROT (Figure 1D). ROT treatment significantly increased the percentage (%) of apoptosis cells in a dose-dependent manner, while Glu/Mal exposure showed no effect on apoptosis (Figure 1E).

### 3.2. Cr(VI) Induced Mitochondria-Related Cytotoxicity

**Figure 2:**
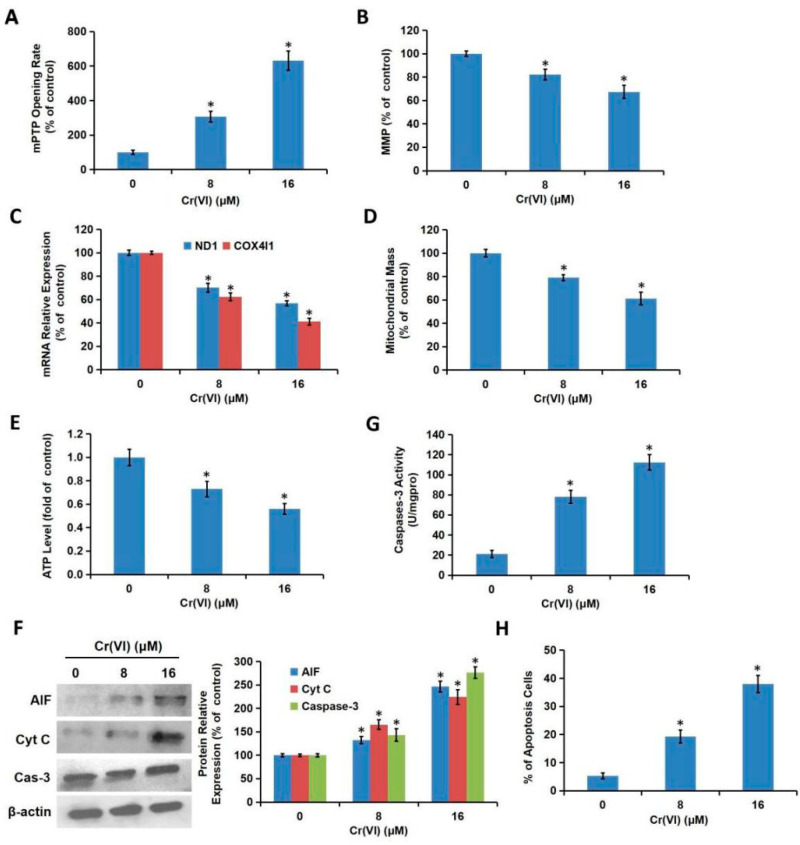
Cr(VI) induced mitochondrial-related cytotoxicity. L02 hepatocytes were treated with different concentrations of Cr(VI) (0, 8, 16 μM) for 24 h. (**A**) The mPTP opening was examined using the commercial kit. (**B**) The MMP was detected using JC-1. (**C**) The mRNA levels of ND1 and COX4I1, which could reflect the copy number of mtDNA were detected using quantitative real-time polymerase chain reaction (qRT-PCR). (**D**) Mitochondria mass was examined using Mito-Tracker Green by flow cytometer. (**E**) The ATP level was examined using a luciferase-based luminescence enhanced ATP assay kit. (**F**) AIF, Cyt C, and caspase-3 protein expressions were detected using Western blotting analysis. The protein bands were quantitated using Image J software. (**G**) Caspase-3 activity was determined using the commercial colorimetric assay kit. (**H**) The cell apoptosis was determined using commercial Annexin V-FITC Apoptosis Detection Kit. * *p* < 0.05, compared with the control group.

It is well known that mPTP and MMP are important parameters reflecting mitochondrial function. The cells were exposed to various concentrations of Cr(VI) (0, 8, 16 μM) for 24 h. Treatment with Cr(VI) caused a marked increase in mPTP opening rate (Figure 2A) and a decrease in MMP (Figure 2B) in a concentration-dependent manner, suggesting the occurrence of mitochondrial damage. The mtDNA copy number and mitochondrial mass, which might be altered during mitochondrial damage, were also detected. mtDNA encodes 13 proteins which closely related to mitochondrial function. We designed the primers for two genes encoded by mtDNA, MRCC I subunit, ND1, and MRCC IV subunit, COX4I1. The expression amount of the two genes can reflect the copy number of mtDNA. As shown in Figure 2C, both ND1 and COX4I1 mRNA levels were decreased after Cr(VI) treatment, indicating the decline of mtDNA copy number. Cr(VI) exposure also decreased mitochondrial mass (Figure 2D). Mitochondria are energy providers, generating more than 95% of the ATP required for cells. Mitochondrial damage results in the decline of the intracellular ATP level, as confirmed in Figure 2E. The mPTP opening marks the emergence of irreversible point of apoptosis, which is accompanied by the release of apoptosis-inducing factors such as AIF and Cyt C from mitochondria to the cytoplasm. Cr(VI) increased the protein levels of AIF and Cyt C (Figure 2F). As a major member of cysteinyl aspartate-specific protease (caspase) family, caspase-3 plays a vital role in apoptosis. Both the protein expression (Figure 2F) and activity (Figure 2G) of caspase-3 were enhanced by Cr(VI) treatment. Cr(VI) also increased the % of apoptosis cells in a concentration-dependent manner (Figure 2H).

### 3.3. Cr(VI) Targeted MRCC I to Induce Cytotoxicity

**Figure 3:**
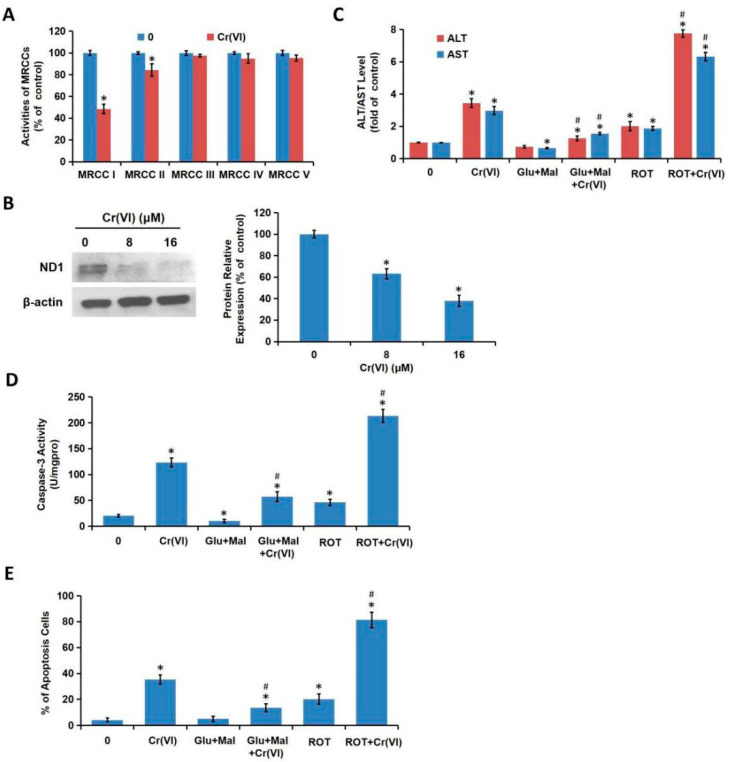
Cr(VI) targeted MRCC I to induce cytotoxicity. (**A**) L02 hepatocytes were treated with different concentrations of Cr(VI) (0, 8, 16 μM) for 24 h. The activities of MRCC I-V were determined spectrophotometrically using the commercial kits. (**B**) The protein expression of ND1 was determined using Western blotting. (**C**) L02 hepatocytes were exposed to Cr(VI) (16 μM) or PBS for 24 h with or without the pretreatment of Glu/Mal (10/10 mM) for 1 h; or the cells were exposed to Cr(VI) (16 μM) or PBS with or without the cotreatment of ROT (5 μM) for 24 h. AST/ALT level was determined using the commercial kits. (**D**) Caspase-3 activity was determined using the commercial colorimetric assay kit. (**E**) The cell apoptosis was determined using commercial Annexin V-FITC Apoptosis Detection Kit. * *p* < 0.05, compared with the control group. # *p* < 0.05, compared with the Cr(VI) alone treatment group.

We have confirmed that MRCC I inhibition led to cytotoxicity in L02 hepatocytes. Next, we verified whether Cr(VI) targeted MRCC I to induce cytotoxicity. L02 hepatocytes were treated with different concentrations of Cr(VI) (0, 8, 16 μM) for 24 h. As shown in Figure 3A, among the five complexes, the activity of MRCC I was significantly decreased while the activity of MRCC II was slightly decreased after Cr(VI) exposure. The protein expression of MRCC I subunit ND1 was also decreased by Cr(VI) in a concentration-dependent manner (Figure 3B). For the combination treatments, L02 hepatocytes were exposed to Cr(VI) (16 μM) or PBS for 24 h with or without the pretreatment of Glu/Mal (10/10 mM) for 1 h; the cells were exposed to Cr(VI) (16 μM) or PBS with or without the cotreatment of ROT (5 μM) for 24 h. The pretreatment of Glu/Mal alleviated Cr(VI)-induced increase of ALT/AST leakage, while the cotreatment of ROT significantly aggravated Cr(VI)-induced increase of ALT/AST leakage (Figure 3C). We then detected the effect of MRCC I on Cr(VI)-induced caspase-3 activation and apoptosis induction. As shown in Figure 3D,E, the pretreatment of Glu/Mal significantly alleviated Cr(VI)-induced caspase-3 activation and apoptosis induction, while the cotreatment of ROT obviously aggravated Cr(VI)-induced caspase-3 activation and apoptosis induction. The inhibiting effect of ROT on MRCC I was more obvious than the enhancing effect of Glu/Mal.

### 3.4. Cr(VI) Induced Mitochondrial Hyper-Fission via Interfering with Drp1

**Figure 4:**
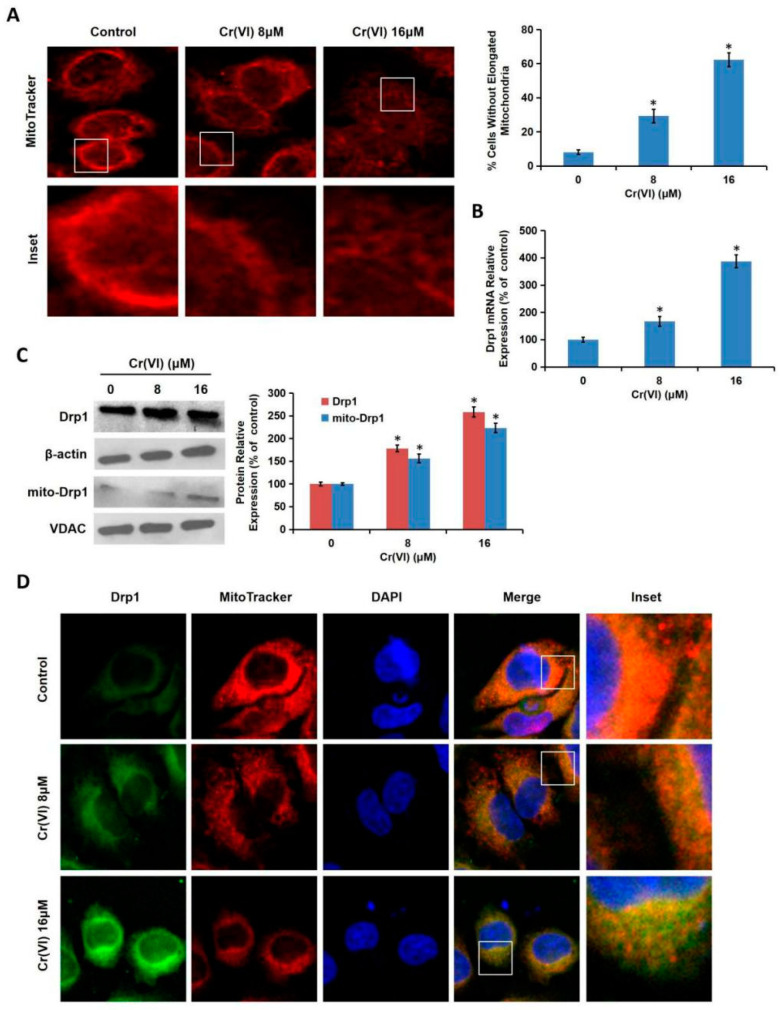
Cr(VI) caused mitochondrial fragmentation. L02 hepatocytes were treated with different concentrations of Cr(VI) (0, 8, 16 μM) for 24 h. (**A**) Mitochondrial morphology was determined using Mitotracker Red by confocal microscope. The % of cells without elongated mitochondria was calculated. (**B**) Drp1 mRNA expression was determined using qRT-PCR. (**C**) Total and mitochondrial Drp1 protein expressions were detected using Western blotting analysis. Voltage-dependent anion channel 1 (VDAC1) served as the loading control of mitochondrial protein. (**D**) Drp1 mitochondrial translocation was observed under a confocal microscope. * *p* < 0.05, compared with the control group.

Drp1, a major protein regulating mitochondrial fission in mammals, acts as an important intrinsic factor involved in mitochondria-dependent apoptosis. We then examined whether Drp1 and Drp1-related mitochondrial fission were involved in Cr(VI)-induced apoptosis. L02 hepatocytes were treated with different concentrations of Cr(VI) (0, 8, 16 μM) for 24 h. As shown in Figure 4A, the mitochondria of the control group revealed the significant large, tubular network structure, while the mitochondria of the Cr(VI)-exposed group showed the short-shaped, divided, and segmented structure, indicating that Cr(VI) induced excessive mitochondrial fragmentation. The % of cells without elongated mitochondria was also shown. Drp1 mRNA expression levels were also increased after Cr(VI) exposure in a concentration-dependent manner, indicating that Cr(VI) caused the transcriptional alteration of Drp1 (Figure 4B). Cr(VI) up-regulated the protein expressions of both the total and mitochondrial Drp1, suggesting that in addition to inducing the translational change of Drp1, Cr(VI) also triggered the translocation of Drp1 from cytoplasm to mitochondria (Figure 4C). The mitochondrial Drp1 translocation was then observed with laser confocal microscopy. As shown in Figure 4D, Drp1 protein with a green dot-like distribution was dramatically increased after Cr(VI) treatment, and the translocation of the increased Drp1 protein to mitochondria was clearly observed. 

**Figure 5:**
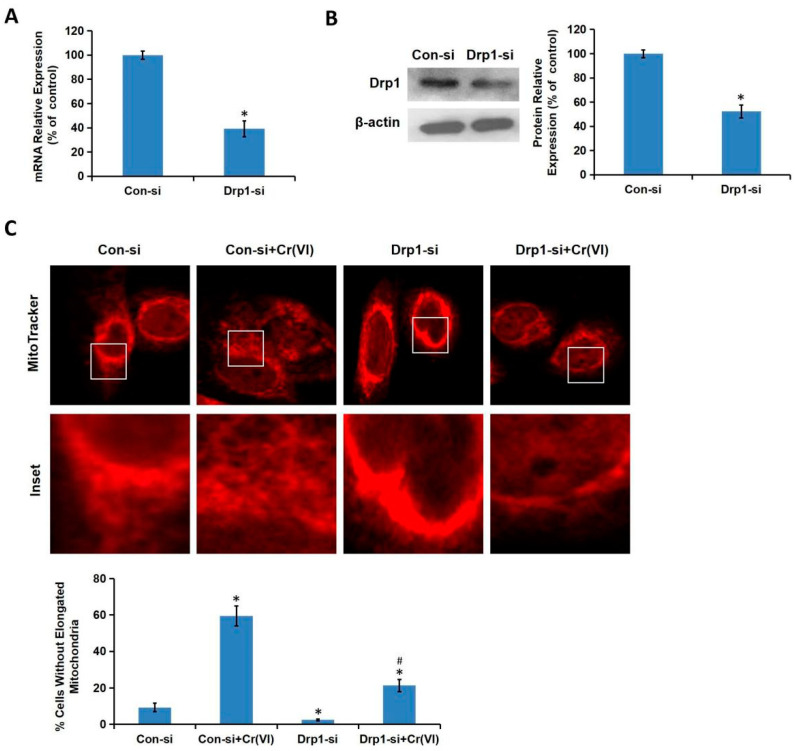
Cr(VI) induced mitochondrial hyper-fission via interfering with Drp1. Drp1 siRNA plasmid was constructed and verified using qRT-PCR (**A**) and Western blotting (**B**). (**C**) The L-02 hepatocytes transfected with Drp1-siRNA and its control (Con-si) were treated with Cr(VI) (16 μM) or PBS for 24 h. Mitochondrial morphology was determined using Mitotracker Red by confocal microscope. The % of cells without elongated mitochondria was calculated. * *p* < 0.05, compared with the control group. # *p* < 0.05, compared with the Con-si plus Cr(VI) treatment group.

We then explored the effect of Drp1-siRNA on Cr(VI)-induced fragmentation. We constructed Drp1-siRNA plasmid and confirmed its efficiency using qRT-PCR (Figure 5A) and Western blotting (Figure 5B). The L-02 hepatocytes transfected with Drp1-siRNA and its control (Con-si) were treated with Cr(VI) (16 μM) or PBS for 24 h. While the significantly short-shaped, divided, and segmented mitochondria were observed in the Cr(VI) exposure group, Drp1-siRNA alleviated the excessive mitochondrial fragmentation and partially restored the large and tubular network structure of mitochondria (Figure 5C).

### 3.5. Drp1-Mediated Mitochondrial Fragmentation Contributes to Cr(VI)-Induced MRCC I-Dependent Cytotoxicity

**Figure 6:**
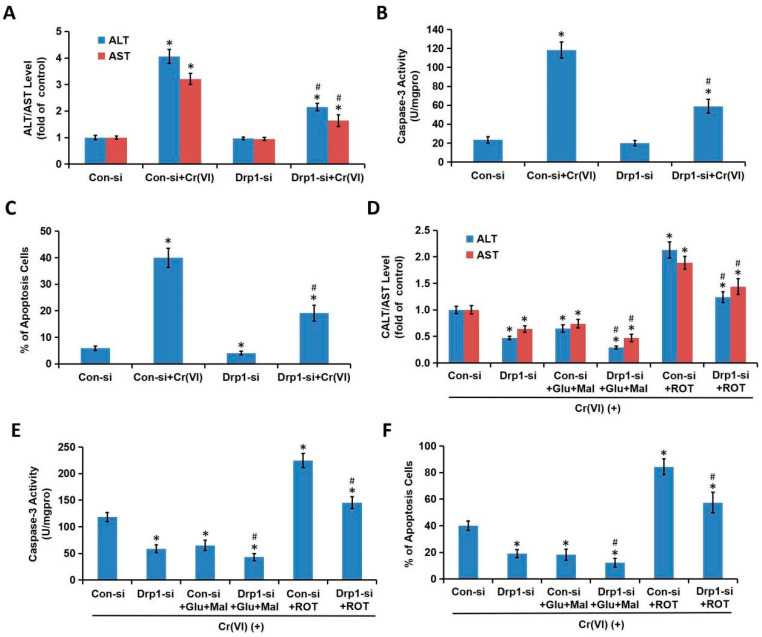
Drp1-mediated mitochondrial fragmentation contributes to Cr(VI)-induced MRCC I-dependent cytotoxicity. (**A**) The L-02 hepatocytes transfected with Drp1-siRNA and Con-si were treated with Cr(VI) (16 μM) or PBS for 24 h. AST/ALT level was determined using the commercial kits. (**B**) Caspase-3 activity was determined using the commercial colorimetric assay kit. (**C**) The cell apoptosis was determined using commercial Annexin V-FITC Apoptosis Detection Kit. (**D**) For the combination treatments, the hepatocytes transfected with Drp1-siRNA and Con-si were exposed to Cr(VI) (16 μM) for 24 h with or without the pretreatment of Glu/Mal (10/10 mM) for 1 h; the hepatocytes transfected with Drp1-siRNA and Con-si were exposed to Cr(VI) (16 μM) with or without the cotreatment of ROT (5 μM) for 24 h. AST/ALT level was determined using the commercial kits. (**E**) Caspase-3 activity was determined using the commercial colorimetric assay kit. (**F**) The cell apoptosis was determined using commercial Annexin V-FITC Apoptosis Detection Kit. * *p* < 0.05, compared with its relative control. # *p* < 0.05, compared with the Drp1-si plus Cr(VI) treatment group.

The effect of Drp1-siRNA on Cr(VI)-induced MRCC I-dependent cytotoxicity was further explored. The L-02 hepatocytes transfected with Drp1-siRNA and Con-si were treated with Cr(VI) (16 μM) or PBS for 24 h. The application of Drp1-siRNA alleviated Cr(VI)-induced ALT/AST leakage (Figure 6A), caspase-3 activation (Figure 6B), and apoptosis induction (Figure 6C). For the combination treatments, the hepatocytes transfected with Drp1-siRNA and Con-si were exposed to Cr(VI) (16 μM) for 24 h with or without the pretreatment of Glu/Mal (10/10 mM) for 1 h; the hepatocytes transfected with Drp1-siRNA and Con-si were exposed to Cr(VI) (16 μM) with or without the cotreatment of ROT (5 μM) for 24 h. As shown in Figure 6D,F, the ALT/AST leakage, caspase-3 activation, and apoptosis induction in Drp1-si plus Cr(VI) treatment group was alleviated by the application of Glu/Mal, and aggravated by the application of ROT. Drp1 siRNA can further promote the inhibition of Glu/Mal on Cr(VI)-induced cytotoxicity, and also can further alleviate the aggravation of ROT on Cr(VI)-induced cytotoxicity.

### 3.6. Cr(VI)-Induced Drp1 Modulation was Depend on MRCC I Inhibition-Mediated ROS Production

**Figure 7:**
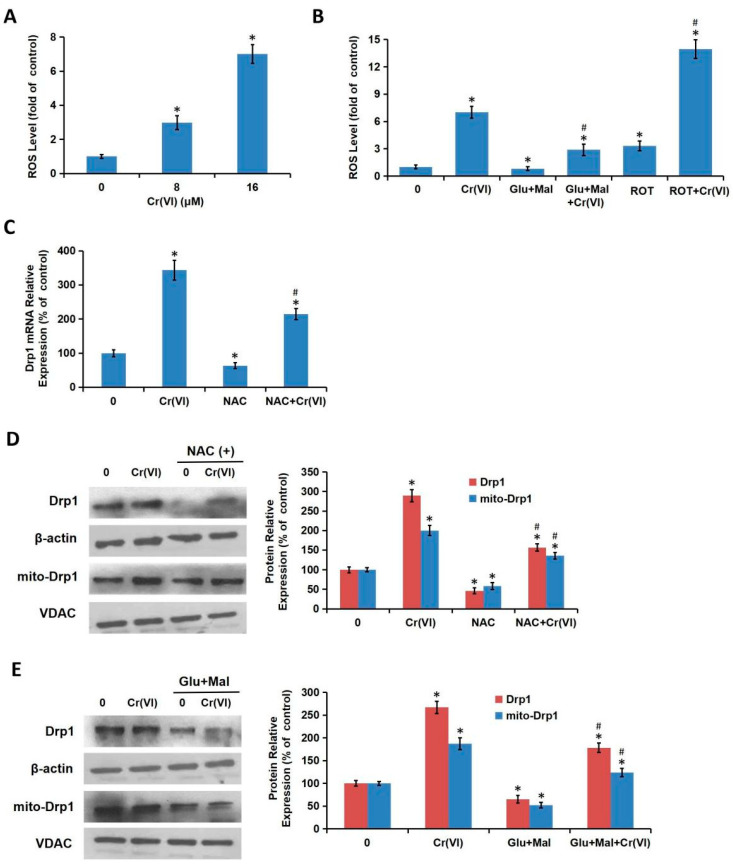
Cr(VI)-induced Drp1 modulation was dependent on MRCC I inhibition-mediated ROS production. (**A**) L02 hepatocytes were treated with different concentrations of Cr(VI) (0, 8, 16 μM) for 24 h. ROS level was determined using the fluorescent probe DCFH-DA. (**B**) L02 hepatocytes were exposed to Cr(VI) (16 μM) or PBS for 24 h with or without the pretreatment of Glu/Mal (10/10 mM) for 1 h; or the cells were exposed to Cr(VI) (16 μM) or PBS with or without the cotreatment of ROT (5 μM) for 24 h. ROS level was determined using the fluorescent probe DCFH-DA. (**C**) The hepatocytes were exposed to Cr(VI) (16 μM) or PBS for 24 h with or without the pretreatment of NAC (5 mM) for 1 h. Drp1 mRNA expression was determined using qRT-PCR. (**D**,**E**) Total and mitochondrial Drp1 protein expressions were detected using Western blotting analysis. * *p* < 0.05, compared with the control group. # *p* < 0.05, compared with the Cr(VI) alone treatment group.

Evidence suggested that oxidative stress could activate Drp1 and promote mitochondrial fragmentation, thus we also explored whether Cr(VI)-induced accumulation of ROS could regulate Drp1. L02 hepatocytes were treated with different concentrations of Cr(VI) (0, 8, 16 μM) for 24 h. As shown in Figure 7A, Cr(VI) caused a significant increase of intracellular ROS levels in a concentration-dependent manner. The hepatocytes were exposed to Cr(VI) or PBS for 24 h with or without the pretreatment of Glu/Mal; or the cells were exposed to Cr(VI) or PBS with or without the cotreatment of ROT. Glu/Mal slightly decreased ROS level compared with the control. Cr(VI)-induced over-production of ROS was alleviated by the application of Glu/Mal, and aggravated by the application of ROT (Figure 7B), indicating that MRCC I was involved in ROS accumulation induced by Cr(VI). The hepatocytes were exposed to Cr(VI) or PBS for 24 h with or without the pretreatment of NAC for 1 h. NAC partially decreased Cr(VI)-induced increase of the mRNA level (Figure 7C), and both the total and mitochondrial protein level (Figure 7D) of Drp1, confirming that Drp1 can be regulated by ROS in both transcription and translation levels. The utilization of Glu/Mal also alleviated the increase of both total and mitochondrial Drp1 expression (Figure 7E). The above results together suggested that Cr(VI)-induced Drp1 modulation was dependent on MRCC I inhibition-mediated ROS production.

## 4. Discussion

We demonstrated in the present study that MRCC I inhibition led to cytotoxicity in L02 hepatocytes, which was characterized by the increase of ALT/AST leakage, the activation of caspase-3, the inhibition of cell proliferation, and the induction of apoptosis. We then confirmed that Cr(VI) targeted MRCC I to induce ROS accumulation and then triggered mitochondrial-related cytotoxicity. Cr(VI) caused a marked increase in mPTP opening rate and decrease in MMP, declined the mtDNA copy number, mitochondrial mass, and ATP level, increased the mitochondrial release of apoptosis-inducing factors, and eventually triggered apoptosis. The pretreatment of Glu/Mal significantly alleviated Cr(VI)-induced cytotoxicity, while the cotreatment of ROT obviously aggravated Cr(VI)-induced cytotoxicity.

In theory, all four complexes (I–IV) of the ETC could generate ROS; evidence suggests that MRCC I and III are the main sites of ROS formation [13,14]. However, the ability of MRCC III to produce ROS has also been questioned, while MRCC I is considered to be the relevant major site of superoxide formation. It is reported that the formation of ROS can not be detected until MRCC III was inhibited by up to 71 ± 4%; in contrast, the small-extent deactivation of MRCC I (16 ± 2%) could lead to a significant increase of ROS generation [15]. It is also demonstrated that the production rate of endogenous H_2_O_2_ was much lower when Glu/Mal, the substrates of MRCC I were used as respiratory fuel; the application of ROT, the inhibitor of MRCC I, led to a significantly increasing H_2_O_2_ release [16]. ROT can interrupt the transfer of electrons to CoQ and enhance ROS production. The amount of ROS produced by various stimulation determines whether ROS play profitable or detrimental roles. In the physiological state, it was believed that ROS appear to be important second messengers that mediate different cell signaling pathways, while the overburdened ROS are exclusively harmful to the cells [17]. The burst generation of ROS leads to irreversible damage to mitochondria, DNA damage, lipid peroxidation, ATP exhaustion, and, eventually, cell death [18]. Although the precise mechanisms involved in ROS burst-generation and ROS-related cytotoxicity are still not clear, the application of specific ROS inhibitors such as NAC and Trolox to reduce the over-production of ROS under pathological conditions has been shown to ameliorate various diseases mediated by oxidative stress [19].

The mtDNA copy number in each mitochondrion is constant, thus, the total number of mtDNA copies reflects the total number of mitochondria in cells [20]. mtDNA is located near the ETC and its repair mechanism is incomplete, thus, mtDNA is more susceptible to damage when exposed to oxidative stress compared with the nuclear DNA [21]. The opening of mPTP represents the abrupt change in permeability of the mitochondrial inner membrane, allowing not only protons but also various ions and solutes of up to 1.5 kDa in size to freely pass through the membrane [22]. Apoptosis is initiated by the change of mitochondrial membrane permeability transition (MPT), and the opening of the non-specific mega-channel mPTP enables the membrane permeability suddenly increase, resulting in the release of the apoptosis executors including apoptosis-inducing factor (AIF), cytochrome c (Cyt c), and endonuclease G from the mitochondrial matrix to cytoplasm. The increase of mPTP opening may lead to the flow back of protons from mitochondrial membrane space to matrix, thus inhibiting MMP and ATP production, resulting in metabolic abnormalities and cytotoxicity [23]. Moreover, Cr(VI) exposure triggered over-opening of mPTP, dropped MMP, decreased mtDNA number, declined ATP synthesis, indicating that Cr(VI) can directly disrupt the structure and function of mitochondria and induce cytotoxicity. As one of the early events of apoptosis induced by various stimuli, the increase of intracellular ROS can trigger Cyt c first detaches from cardiolipin and then being released into the cytoplasm. Cr(VI) exposure disrupted mitochondrial redox homeostasis and accumulated excessive ROS, then mitochondrial function such as MMP and mPTP was severely affected, thus, L02 hepatocytes undergo mitochondria-mediated apoptosis.

Cr(VI) induced excessive mitochondrial fragmentation and mitochondrial Drp1 translocation, the application of Drp1-siRNA alleviated Cr(VI)-induced ALT/AST leakage, caspase-3 activation, and apoptosis induction. The cytotoxicity in Drp1-si plus Cr(VI) treatment group was alleviated by the application of Glu/Mal, and aggravated by the application of ROT. Drp1 siRNA can further promote the inhibition of Glu/Mal on Cr(VI)-induced cytotoxicity, and also can further alleviate the aggravation of ROT on Cr(VI)-induced cytotoxicity. The dynamic network of mitochondria can meet the energy and metabolic needs of cells. The intracellular mitochondrial morphology network represents a perfect balance between the fusion/fission events [24]. Mitochondrial dynamics is mainly regulated by Drp1, mitofusin (Mfn) 1 and Mfn2, and other mitochondrial fusion and fission-related proteins [25]. Once the damage progressed to the irreversible stage, mitochondria will reveal excessive fission and fragmentation, mass decrease, as well as membrane integrity loss. The application of Drp1 siRNA or Mdivi-1 (a specific Drp1 inhibitor) can inhibit the conversion to punctate mitochondrial phenotype, weaken the insertion and oligomerization of pro-apoptotic Bax protein, thus attenuating cell apoptosis, reducing transient focal ischemia-induced infarct and neurological deficits. Drp1 activity could be altered under all kinds of stimulation, leading to mitochondrial dynamics abnormality and cell damage [26]. It is reported that oxidative stress can enhance the activity of Drp1 and promote mitochondrial fragmentation and dysfunction mediated by Drp1, while the application of antioxidants can restore mitochondrial morphology [27,28]. Evidence suggested that the upstream transcription activator of Drp1 is p53, which could promote Drp1 transcription by binding to its promoter [29]. In the present study, we confirmed that Cr(VI)-induced Drp1 modulation was dependent on MRCC I inhibition-mediated ROS production. Taken together, this study demonstrated that Drp1-mediated mitochondrial fragmentation contributes to Cr(VI)-induced MRCC I-dependent cytotoxicity.

## 5. Conclusions

**Figure 8:**
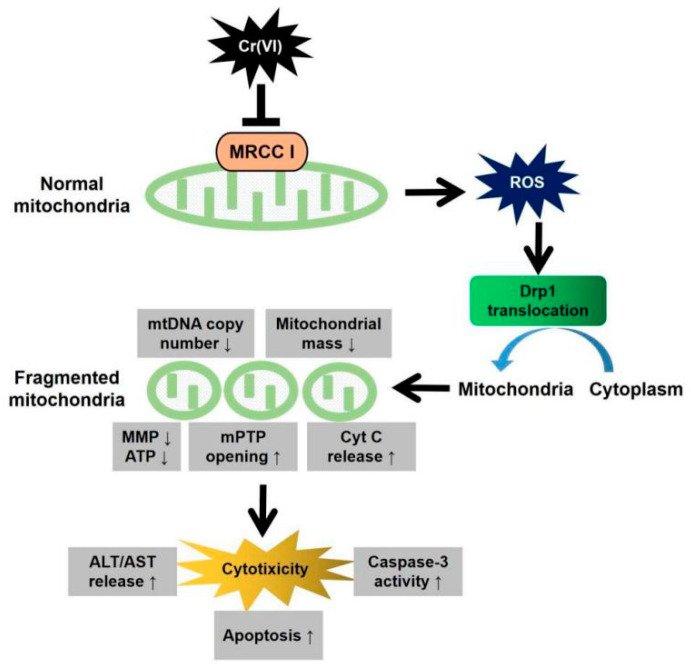
Summary chart of the research content of this paper. Increased mitochondrial fragmentation mediated by Drp1 contributes to Cr(VI)-induced MRCC I-dependent cytotoxicity.

In summary, Cr(VI) treatment inhibited the activity of MRCC I and enhanced ROS generation in L02 hepatocytes, leading to the translocation of Drp1 from the cytoplasm to mitochondria, subsequently inducing excessive fission and fragmentation of mitochondria. The resulted imbalance of mitochondrial dynamics decreased mtDNA copy number and mitochondrial mass, and then impaired mitochondrial function, mainly manifestation as the collapse of MMP, the exhaustion of ATP, the abnormal opening of mPTP, and the mitochondrial release of apoptosis-inducing factors such as AIF and Cyt c. Eventually, Cr(VI)-cytotoxicity was characterized by the increase of ALT/AST leakage, the enhancement of caspase-3 activity, and apoptosis (Figure 8). In this process, it is not clear whether phosphorylation or other modifications of Drp 1 occurs, and this is also our next research direction.

## References

[1] Chebeir M., Liu H. (2018). Oxidation of cr(iii)-fe(iii) mixed-phase hydroxides by chlorine: Implications on the control of hexavalent chromium in drinking water. Environ. Sci. Technol..

[2] Gong K., Qian H., Lu Y., Min L., Guo Z. (2018). Ultrasonic pretreated sludge derived stable magnetic active carbon for cr(vi) removal from wastewater. ACS Sustain. Chem. Eng..

[3] Chandhok G., Lazarou M., Neumann B. (2018). Structure, function, and regulation of mitofusin-2 in health and disease. Biol. Rev..

[4] Wang P., Li Y., Yang Z., Yu T., Tang W. (2018). Inhibition of dynamin-related protein 1 has neuroprotective effect comparable to therapeutic hypothermia in a rat model of cardiac arrest. Transl. Res..

[5] Brooks C., Wei Q., Feng L., Dong G., Tao Y., Mei L., Xie Z.-J., Dong Z. (2007). Bak regulates mitochondrial morphology and pathology during apoptosis by interacting with mitofusins. Proc. Natl. Acad. Sci. USA.

[6] Ishihara N., Nomura M., Jofuku A., Kato H., Suzuki S.O., Masuda K., Otera H., Nakanishi Y., Nonaka I., Goto Y.-I. (2009). Mitochondrial fission factor drp1 is essential for embryonic development and synapse formation in mice. Nat. Cell Biol..

[7] Wakabayashi J., Zhang Z., Wakabayashi N., Tamura Y., Sesaki H. (2009). The dynamin-related gtpase drp1 is required for embryonic and brain development in mice. J. Cell Biol..

[8] Beutner G., Porter G.A. (2017). Analyzing supercomplexes of the mitochondrial electron transport chain with native electrophoresis, in-gel assays, and electroelution. J. Vis. Exp..

[9] Jones A.J.Y., Blaza J.N., Varghese F., Hirst J. (2017). Respiratory complex i in bos taurus and paracoccus denitrificans pumps four protons across the membrane for every nadh oxidized. J. Biol. Chem..

[10] Cadenas E., Davies K.J.A. (2000). Mitochondrial free radical generation, oxidative stress, and aging 1 1 this article is dedicated to the memory of our dear friend, colleague, and mentor lars ernster (1920–1998), in gratitude for all he gave to us. Free Radic. Biol. Med..

[11] Xiao Y., Zeng M., Yin L., Li N., Xiao F. (2019). Clusterin increases mitochondrial respiratory chain complex i activity and protects against hexavalent chromium-induced cytotoxicity in l-02 hepatocytes. Toxicol. Res..

[12] Zhang Y., Zhang Y., Xiao Y., Zhong C., Xiao F. (2019). Expression of clusterin suppresses cr(vi)-induced premature senescence through activation of pi3k/akt pathway. Ecotoxicol. Environ. Saf..

[13] Potargowicz E., Szerszenowicz E., Staniszewska M., Nowak D. (2005). Mitochondria as a source of reactive oxygen species. Postepy Hig Med. Dosw..

[14] Landazabal M.A.B., Otero A.L.C., Kouznetsov V.V., Duque J.E., Mendez-Sanchez S.C. (2018). Alterations of mitochondrial electron transport chain and oxidative stress induced by alkaloid-like α-aminonitriles on aedes aegypti larvae. Pestic. Biochem. Physiol..

[15] Sipos I., Tretter L., Adam-Vizi V. (2002). Quantitative relationship between inhibition of respiratory complexes and formation of reactive oxygen species in isolated nerve terminals & nbsp. J. Neurochem..

[16] Ohnishi S.T., Shinzawa-Itoh K., Ohta K., Yoshikawa S., Ohnishi T. (2010). New insights into the superoxide generation sites in bovine heart nadh-ubiquinone oxidoreductase (complex i): The significance of protein-associated ubiquinone and the dynamic shifting of generation sites between semiflavin and semiquinone radicals. Biochim. Biophys. Acta.

[17] Brand M.D. (2016). Mitochondrial generation of superoxide and hydrogen peroxide as the source of mitochondrial redox signaling. Free Radic. Biol. Med..

[18] Orrenius S., Gogvadze V., Zhivotovsky B. (2007). Mitochondrial oxidative stress: Implications for cell death. Annu. Rev. Pharmacol. Toxicol..

[19] Ahmad W., Ijaz B., Shabbiri K., Ahmed F., Rehman S. (2017). Oxidative toxicity in diabetes and alzheimer’s disease: Mechanisms behind ros/rns generation. J. Biomed. Sci..

[20] Medeiros T.C., Graef M. (2018). Autophagy determines mtdna copy number dynamics during starvation. Autophagy.

[21] Herbers E., Kekäläinen N.J., Hangas A., Pohjoismäki J.L., Goffart S. (2018). Tissue specific differences in mitochondrial DNA maintenance and expression. Mitochondrion.

[22] Karch J., Kwong J.Q., Burr A.R., Sargent M.A., Elrod J.W., Peixoto P.M., Martinez-Caballero S., Osinska H., Cheng H.Y., Robbins J. (2013). Bax and bak function as the outer membrane component of the mitochondrial permeability pore in regulating necrotic cell death in mice. Elife.

[23] Ren D.D., Sun J., Chen D., Gao J., Amp M., Laboratory N., Pharmacy S.O., University J. (2017). Regulation mechanism and targeted drugs of mitochondrial permeability transition pore on programmed cell death:Research advances. Int. J. Curr. Pharm. Res..

[24] Matsumura A., Higuchi J., Watanabe Y., Kato M., Aoki K., Akabane S., Endo T., Oka T. (2018). Inactivation of cardiolipin synthase triggers changes in mitochondrial morphology. FEBS Lett..

[25] Yoo S.M., Jung Y.K. (2018). A molecular approach to mitophagy and mitochondrial dynamics. Mol. Cells.

[26] Schmitt K., Grimm A., Dallmann R., Oettinghaus B., Restelli L.M. (2018). Circadian control of drp1 activity regulates mitochondrial dynamics and bioenergetics. Cell Metab..

[27] Wu S., Zhou F., Zhang Z., Xing D. (2011). Mitochondrial oxidative stress causes mitochondrial fragmentation via differential modulation of mitochondrial fission-fusion proteins. FEBS J..

[28] Chia-Hua C., Ching-Chih L., Ming-Chang Y., Chih-Chang W., Huei-De L., Run-Chin L., Wen-Yu T., Tsung-Chieh K., Ching-Mei H., Jiin-Tsuey C. (2012). Gsk3beta-mediated drp1 phosphorylation induced elongated mitochondrial morphology against oxidative stress. PLoS ONE.

[29] Yuan Y., Zhang A., Qi J., Wang H., Liu X., Zhao M., Duan S., Huang Z., Zhang C., Wu L. (2018). P53/drp1-dependent mitochondrial fission mediates aldosterone-induced podocyte injury and mitochondrial dysfunction. Am. J. Physiol. Ren. Physiol..

